# Metabolome patterns identify active dechlorination in bioaugmentation consortium SDC-9™

**DOI:** 10.3389/fmicb.2022.981994

**Published:** 2022-10-25

**Authors:** Amanda L. May, Yongchao Xie, Fadime Kara Murdoch, Mandy M. Michalsen, Frank E. Löffler, Shawn R. Campagna

**Affiliations:** ^1^Center for Environmental Biotechnology, University of Tennessee, Knoxville, TN, United States; ^2^Department of Civil and Environmental Engineering, Tickle College of Engineering, University of Tennessee, Knoxville, TN, United States; ^3^Environmental Laboratory, U.S. Army Engineer Research and Development Center, Vicksburg, MS, United States; ^4^Department of Microbiology, College of Arts and Sciences, The University of Tennessee, Knoxville, TN, United States; ^5^Department of Biosystems Engineering and Soil Science, Herbert College of Agriculture, The University of Tennessee, Knoxville, TN, United States; ^6^Oak Ridge National Laboratory, Biosciences Division, Oak Ridge, TN, United States; ^7^Department of Chemistry, College of Arts and Sciences, The University of Tennessee, Knoxville, TN, United States; ^8^Biological and Small Molecule Mass Spectrometry Core, College of Arts and Sciences, The University of Tennessee, Knoxville, TN, United States; ^9^University of Tennessee-Oak Ridge Innovation Institute, University of Tennessee, Knoxville, TN, United States

**Keywords:** metabolomics, consortium SDC-9™, bioremediation, reductive dechlorination, environmental monitoring, *Dehalococcoidia*, biomarkers

## Abstract

Ultra-high performance liquid chromatography–high-resolution mass spectrometry (UPHLC–HRMS) is used to discover and monitor single or sets of biomarkers informing about metabolic processes of interest. The technique can detect 1000’s of molecules (i.e., metabolites) in a single instrument run and provide a measurement of the global metabolome, which could be a fingerprint of activity. Despite the power of this approach, technical challenges have hindered the effective use of metabolomics to interrogate microbial communities implicated in the removal of priority contaminants. Herein, our efforts to circumvent these challenges and apply this emerging systems biology technique to microbiomes relevant for contaminant biodegradation will be discussed. Chlorinated ethenes impact many contaminated sites, and detoxification can be achieved by organohalide-respiring bacteria, a process currently assessed by quantitative gene-centric tools (e.g., quantitative PCR). This laboratory study monitored the metabolome of the SDC-9™ bioaugmentation consortium during *cis*-1,2-dichloroethene (cDCE) conversion to vinyl chloride (VC) and nontoxic ethene. Untargeted metabolomics using an UHPLC-Orbitrap mass spectrometer and performed on SDC-9™ cultures at different stages of the reductive dechlorination process detected ~10,000 spectral features per sample arising from water-soluble molecules with both known and unknown structures. Multivariate statistical techniques including partial least squares-discriminate analysis (PLSDA) identified patterns of measurable spectral features (peak patterns) that correlated with dechlorination (in)activity, and ANOVA analyses identified 18 potential biomarkers for this process. Statistical clustering of samples with these 18 features identified dechlorination activity more reliably than clustering of samples based only on chlorinated ethene concentration and *Dhc* 16S rRNA gene abundance data, highlighting the potential value of metabolomic workflows as an innovative site assessment and bioremediation monitoring tool.

## Introduction

Metabolomics (Patti et al., 2012; Viant and Sommer, 2013) has emerged as a powerful technique to monitor microbial processes in biological systems of increasing complexity (Fiehn and Weckwerth, 2003). Specifically, advancements in high-resolution mass spectrometry, such as the Orbitrap mass analyzer, (Fiehn and Weckwerth, 2003; Lu et al., 2010) have allowed to obtain large datasets and generate a more complete picture of the metabolites of a biological system. However, the application of this technique to the monitoring of bioremediation processes is underexplored. Chlorinated solvents, in particular chlorinated ethenes, are common contaminants impacting Superfund sites (Moran et al., 2007) and other groundwater aquifers, and are suspected or proven cancer-causing agents (Huang et al., 2014; Chlorinated Solvents Contamination, 2016). Organohalide-respiring bacteria can detoxify chlorinated ethenes through the stepwise reductive dechlorination to benign ethene (Maymó-Gatell et al., 1997; He et al., 2003; Löffler et al., 2013; Adrian and Löffler, 2016). In this anaerobic respiratory process, chlorinated ethenes are terminal electron acceptors to conserve energy for growth, a process associated with the release of inorganic chloride. Several strains of *Dhc* have been shown to dechlorinate dichloroethene isomers and vinyl chloride (VC) to ethene (Yang et al., 2017) and the key reductive dehalogenase (RDase) genes involved in this process have been identified (Krajmalnik-Brown et al., 2004; Müller et al., 2004; Lee et al., 2006; Löffler et al., 2013; Zinder, 2016; Yan et al., 2021). The commercial bioaugmentation consortium SDC-9™ is utilized as a bioaugmentation culture to stimulate reductive dechlorination of chlorinated ethenes and ethene formation (Löffler and Edwards, 2006). Documenting this process at field sites is not straightforward because of interferences with the quantitative measurements of chlorinated ethenes and ethene. To establish confidence in detoxification, a multiple lines of evidence approach is desirable, including a direct measure of activity of organohalide-respiring *Dhc*.

Molecular biological tools (MBTs) targeting biomarker genes, transcripts, and/or proteins of organohalide-respiring bacteria are employed for site assessment and bioremediation monitoring (Amos et al., 2008; Matturro and Rossetti, 2015; Kanitkar et al., 2017). Quantitative PCR (qPCR) targeting of *Dehalococcoides mccartyi* (*Dhc*) biomarker genes serves as a robust MBT in bioremediation monitoring regimes, and a *Dhc* population exceeding cell abundances of 10^7^ l^−1^ is thought to be sufficiently large to reduce the contaminant concentrations over time periods acceptable for most bioremediation projects (Lu et al., 2006). However, the correlation between *Dhc* gene copy number and the rate of bioremediation does not provide a direct relationship linking the two, (Lee et al., 2006; Silva and Alvarez, 2008; Michalsen et al., 2022) and the rate can be influenced by environmental factors, such as temperature (Fletcher et al., 2011) or oxygen (Amos et al., 2008). To obtain more direct information about dechlorination activity, MBTs targeting *Dhc* biomarker transcripts and proteins have been developed; however, the implementation and robustness of these approaches for *Dhc* monitoring at field sites either have limitations or have yet to be fully validated. These efforts have introduced several biomarkers as possibilities to use monitor the bioremediation strategies associated with the dechlorination process based on the abundances of genes associated with a specific process using qPCR (e.g., *Dhc* VC RDase genes abundances) (Krajmalnik-Brown et al., 2004; Müller et al., 2004; Löffler et al., 2013) or proteomics (e.g., TceA, VcrA, BvcA protein concentrations) (Solis et al., 2019). However, the mere presence of a metabolic gene or protein does not imply that the host is metabolically active.(Amos et al., 2008).

Metabolomics has the ability to circumvent the limitations of other MBTs by providing direct information about metabolic activity, and an increasing number of metabolomics studies in clinical (Gonzalez-Covarrubias et al., 2022), toxicological (Morgan et al., 2022), and environmental (Wei et al., 2022) settings demonstrated the value of this approach (Pang et al., 2021). Bioremediation of chlorinated ethenes is also often hindered by the process stalling, which leaves behind either *cis*-1,2-dichloroethene (cDCE) or vinyl chloride (Lihl et al., 2019). Recognizing sites where cDCE/VC stalls are likely to occur would be advantageous, as it would allow timely implementation of adaptive site management decision-making to predict and possibly prevent stalled dechlorination. Supplementing MBTs based on gene copy number with biomarkers of physiological activity would provide a more nuanced understanding of the bioremediation progress and could also help to predict and avoid stalls. Therefore, this work aimed to use metabolomics to identify a set of metabolite biomarkers that could complement current validated MBTs with indicators for the metabolic activity of the dechlorinating consortium. Such targeted analyses for a small set of molecules could be performed using routine analytical instrumentation and methods analogous to those employed in many environmental monitoring laboratories to measure PFAS concentrations. If metabolomics can be used to monitor this process, a snapshot of the *in situ* metabolic state of the microbiome can be captured and unique profiles/patterns could be identified and associated with *Dhc* reductive dechlorination activity. For this study, it was hypothesized that organohalide-respiring *Dhc* produces characteristic metabolic profiles that could be correlated to the activity of the SDC-9™ consortium, and ultra-high performance liquid chromatography—high resolution mass spectrometry (UHPLC-HRMS) metabolomics was used to measure potential biomarkers for the reductive dechlorination processes.

## Materials and methods

### SDC-9™ consortium growth conditions

The consortium SDC-9™ was provided by APTIM (Lawrenceville, NJ, United States) as a concentrated stock suspension (~10^9^ cells/ml). Glass serum bottles (160 ml) containing 100 ml of bicarbonate-buffered defined mineral salt medium (Löffler et al., 2005) were sealed with rubber stoppers and autoclaved. Once cooled, filter-sterilized vitamin mix (Wolin et al., 1963), lactate (10 mM), and 50 μg/l vitamin B_12_ were added to the medium. Cultures were then inoculated (5% *v/v*) directly from the stock culture suspension inside an anoxic chamber filled with hydrogen (3% *v/v*) and nitrogen (97% *v/v*). The electron acceptor, cDCE (6 μl, 79.2 μmol) or VC gas (2 ml, 83.3 μmol) was added. Control cultures received no electron acceptor. All cultures were incubated at room temperature (22°C) in the dark under static conditions. Entire cultures were sacrificed before initiation of dechlorination (100% cDCE/VC remaining, t0), at the beginning of dechlorination (>90% cDCE/VC remaining, t1), during active dechlorination (20–80% cDCE/VC remaining, t2-3), and for up to 14 days after dechlorination was completed (0% cDCE/VC remaining, t5-7) (Table 1). Control samples (Con) with no added chlorinated ethenes were also collected as indicated in Table 1.

**Table 1:** Time points of sample collection during growth of SDC-9™ with either cDCE or VC.

	SDC-9™ +/− cDCE			SDC-9™ +/− VC		
% cDCE/VC remaining^a^	+ cDCE	cDCE-NA^b^	Days	+ VC	VC-NA^b^	Days
100%	cDCE t0 (3)	^d^	0	VC t0 (5)	^d^	0
>90%	cDCE t1 (5)	Con t1^c^ (3)	0.3	VC t1 (5)	Con t1^c^ (5)	0.3
60–80%	cDCE t2 (5)	^d^	1.1	VC t2 (5)	Con t2 (5)	1.4
20–40%	cDCE t3 (5)	^d^	5.9	VC t3 (5)	Con t3 (5)	6
0%	cDCE t4 (5)	Con t4^c^ (3)	17.8	VC t4 (5)	Con t4^c^ (5)	19.8
5 days after t4	cDCE t5 (5)	^d^	30	VC t5 (5)	Con t5 (5)	25.9
0% VC (cDCE only)	cDCE t6 (3)	^d^	38.9	^d^	^d^	^d^
~25 days after t4	cDCE t7 (5)	Con t7 (3)	52.9	^e^	^e^	47.9^e^

Number of replicates indicated in parentheses.

a % chlorinated ethene remaining (cDCE or VC).

b Control cultures without chlorinated electron acceptor (NA).

c Control cultures collected at the same time point were combined for analysis.

d Sample not collected.

e Samples not included in metabolomics analysis.

### Analysis of chlorinated ethenes and ethene

**Figure 1:**
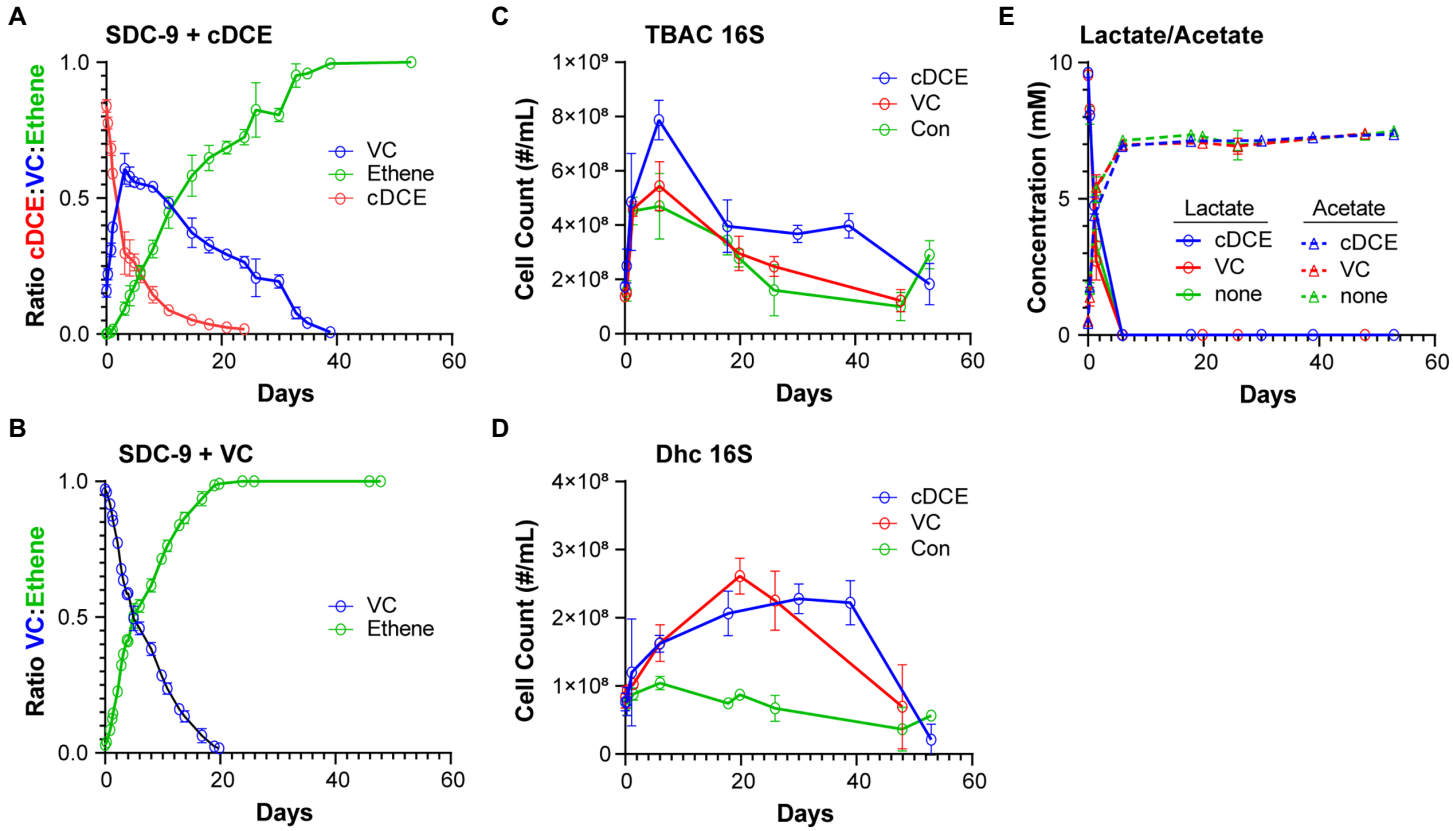
(Chloro)ethene, qPCR, and electron donor data illustrating the performance of SDC-9™ cultures utilizing cDCE or VC as an electron acceptor. (Chloro)ethene concentrations measured during dechlorination of **(A)** cDCE and **(B)** VC to ethene; 16S rRNA gene copy numbers for **(C)** total bacteria (TBAC) and **(D)** *Dhc* in cultures under different growth conditions; and **(E)** lactate consumption and acetate formation in all cultures. Error bars represent standard deviation of replicate samples.

Chlorinated ethenes and ethene were analyzed using an Agilent 7,890 gas chromatograph (Yan et al., 2015). Headspace samples (0.2 ml) were directly injected onto a DB-624 capillary column (60 m × 0.32 mm × 1.8 μm) and detected using flame ionization. As dechlorination proceeded, headspace samples for each culture were taken and subjected to GC analysis to determine the concentrations of cDCE, VC, and ethene. The peak areas for these compounds were integrated and converted to normalized concentrations using a combined standard of known concentrations of cDCE, VC, and ethene. The percent of volatile organic compounds (VOCs) remaining in the headspace of each culture were calculated (Figures 1A,B).

### Lactate and acetate quantification

At each designated time point, aliquots (1 ml) from replicate cultures were taken and stored at −80°C until analysis. Lactate and acetate were quantified with an Agilent 1,200 series HPLC system equipped with an Aminex HPX-87H column (300 mm × 7.8 mm) and a UV detector set to a wavelength of 210 nm. Peak areas were integrated, and concentrations were determined based on an external calibration curve determined from linear regression using an intercept of 0: Peak Area (Lactate) = 175.27 × Lactate Concentration (mM) (regression *R*^2^ = 0.9999) and Peak Area (Acetate) = 77.51 × Acetate Concentration (mM) (regression *R*^2^ = 0.9999) (Figure 1E).

### DNA isolation

At each designated, time point aliquots (1 ml) from replicate cultures were collected on 0.2 μm filters (25 mm, Millipore Durapore membrane filter) and stored at −80°C until analysis. DNA was extracted from each filter using the DNeasy PowerLyzer PowerSoil Kit (Qiagen, Valencia, CA) according to the manufacturer’s instructions. To achieve cell lysis, a bead-beating method using the OMNI Bead Ruptor Homogenizer (OMNI International, GA) at 5 m/s for 3 min was used. DNA was eluted with nuclease-free water and quantified using the Qubit dsDNA HS Assay Kit according to the manufacturer’s recommendations (Invitrogen, Carlsbad, CA). DNA was stored at −80°C until qPCR analysis.

### qPCR analysis

TaqMan qPCR was conducted in 10-μL reaction volumes containing 5 μl of TaqMan Universal PCR Master Mix No AmpErase UNG (Applied Biosystems, Carlsbad, CA), 0.3 μM of each primer, 0.3 μM of probe and 2 μl of DNA template (undiluted and 1:10 diluted). The PCR cycle parameters applied were as follows: 2 min at 50°C and 10 min at 95°C, followed by 40 cycles of 15 s at 95°C and 1 min at 60°C. All qPCR reactions were performed with the QuantStudio 12 K Flex Real-Time PCR System (Applied Biosystems, Carlsbad, CA) using 384-well qPCR plate. All qPCR assays were performed in triplicate and standard curves were included with every qPCR plate using 10-fold serial dilutions of plasmid DNA for each target gene over 7 orders of magnitude ranging from 1 ng/μL (∼8 log gene copies) to 10^−7^ ng/μL. The qPCR assay results were analyzed using the QuantStudio 12 K Flex Real-Time PCR System Software (Applied Biosystems, Carlsbad, CA). Previously established primers and probes were used to enumerate total bacteria and *Dhc* 16S rRNA genes (Figures 1C,D). (Johnson et al., 2005; Ritalahti et al., 2006; Hatt and Löffler, 2012; Hatt et al., 2013).

### Water-soluble metabolite extraction from laboratory samples

This method was adapted from Bennett et al. (2008). Individual replicate cultures taken performance liquid chromatography high-resolution mass spectrometry individual replicate cultures taken from the collection of total cultures were sacrificed to collect data for each time point, and a portion (50 ml) of the contents were collected by vacuum filtration through a polycarbonate membrane filter (47 mm diameter, 0.2 μM pore size). Filters were then placed upside down in a plastic Petri dish (47 mm) containing 1.3 ml of chilled (−20°C) extraction solvent (40:40:20 acetonitrile:methanol:water with 0.1 M formic acid). Extraction was continued for 15–20 min at −20°C at which point the filter was flipped right side up and washed. The extraction solvent was transferred to a 2 ml microcentrifuge tube. The filter was then washed with 200 μl chilled (−20°C) extraction solvent and added to the microcentrifuge tube and the combined extracts were centrifuged (5 min, 13.2 rpm, 4°C). The supernatant from each tube was removed and transferred to a clean vial. The pellet was then extracted with 200 μl of extraction solvent (chilled to −20°C) for 15 min at −20°C, after which the samples were once again centrifuged (5 min, 16.6 kg, 4°C). The supernatants were removed and combined with the previously collected supernatant, and the extraction process was repeated once more. The combined supernatants were then dried under a stream of nitrogen and stored at −80°C until analysis.

### Ultra-high performance liquid chromatography high-resolution mass spectrometry

This method was adapted from Lu et al. (2010). Prior to analysis, samples were reconstituted in 300 μl of sterile MilliQ water, transferred to an autosampler vial, and placed in an autosampler tray kept at 4°C. Ten microliters from each sample was injected through a Synergi 2.5 micron Hydro-RP 100, 100 × 2.00 mm LC column (Phenomenex, Torrance, CA) kept at 25°C and eluted using the following solvents: (A) 97:3 water:methanol, 10 mM tributylamine, and 15 mM acetic acid, (B) methanol; and gradient: *t* = 0 min; 0% B; t = 2.5 min, 0% B; *t* = 5 min, 20% B; *t* = 7.5 min, 20% B; *t* = 13 min, 55% B, *t* = 15.5 min, 95% B; *t* = 18.5 min, 95% B; *t* = 19 min, 0% B; *t* = 25 min, 0% B; using a flow rate of 200 μl/min. The eluent was then introduced into the mass spectrometer (MS) *via* electrospray ionization (ESI) attached to a Thermo Scientific Exactive Plus Orbitrap MS (Waltham, MA). Data were collected in full scan mode with negative ionization mode using a previously established method (Lu et al., 2010). Samples were run with a spray voltage of 3 kV. The nitrogen sheath gas was set to 10 psi, with a capillary temperature of 320°C; and AGC target was set to 3e6. Samples were analyzed with a resolution of 140,000. A scan window of 85 to 800 *m/z* (mass-to-charge) was used from 0 to 9 min, and a window of 110 to 1,000 *m/z* from 9 to 25 min.

### Metabolomics data pre-processing, processing, and analysis

**Figure 2:**
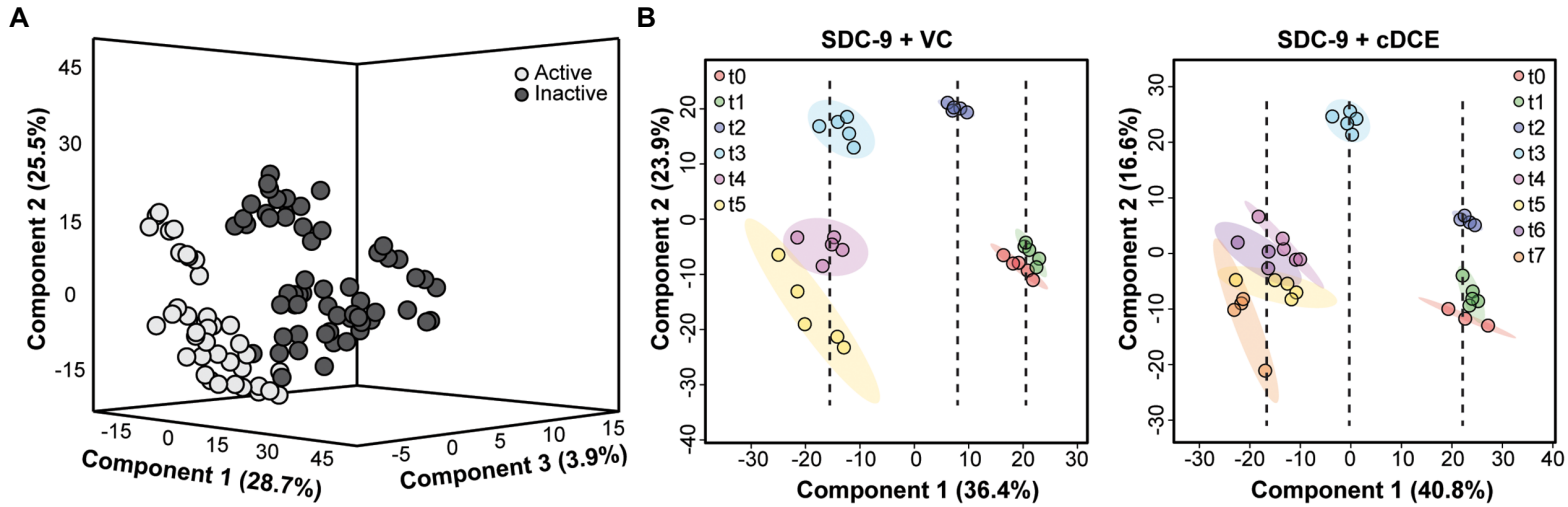
Multivariate analyses of samples classified by dechlorination status or time point. **(A)** 3D PLSDA showing separation between samples classified by dechlorinating activity (Active or Inactive) but not classified by time; **(B)** PLSDA showing separation between samples classified by time point, but not dechlorinating activity, for cultures grown with either VC or cDCE.

**Figure 3:**
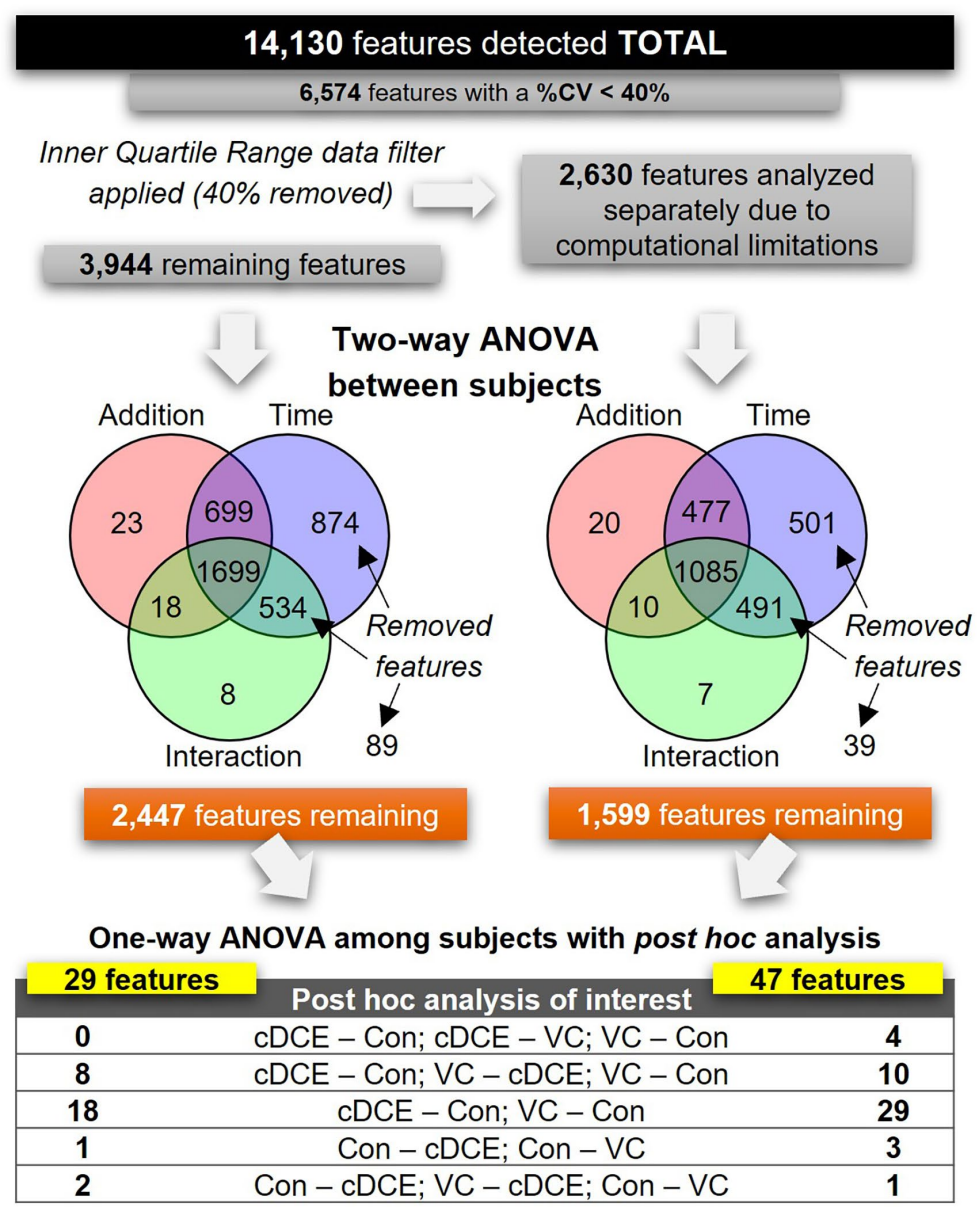
Flowchart outlining data analysis steps for all detected features.

**Figure 4:**
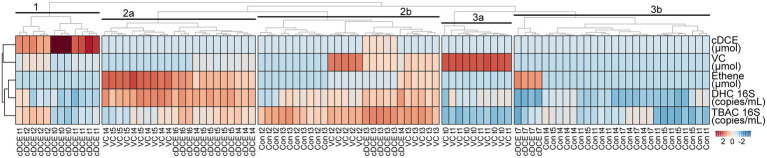
Correlation heatmap of metadata. Abbrev: DHC: *Dehalococcoides*, TBAC: total bacteria, cDCE: *cis*-dichloroethene, VC: vinyl chloride.

**Figure 5:**
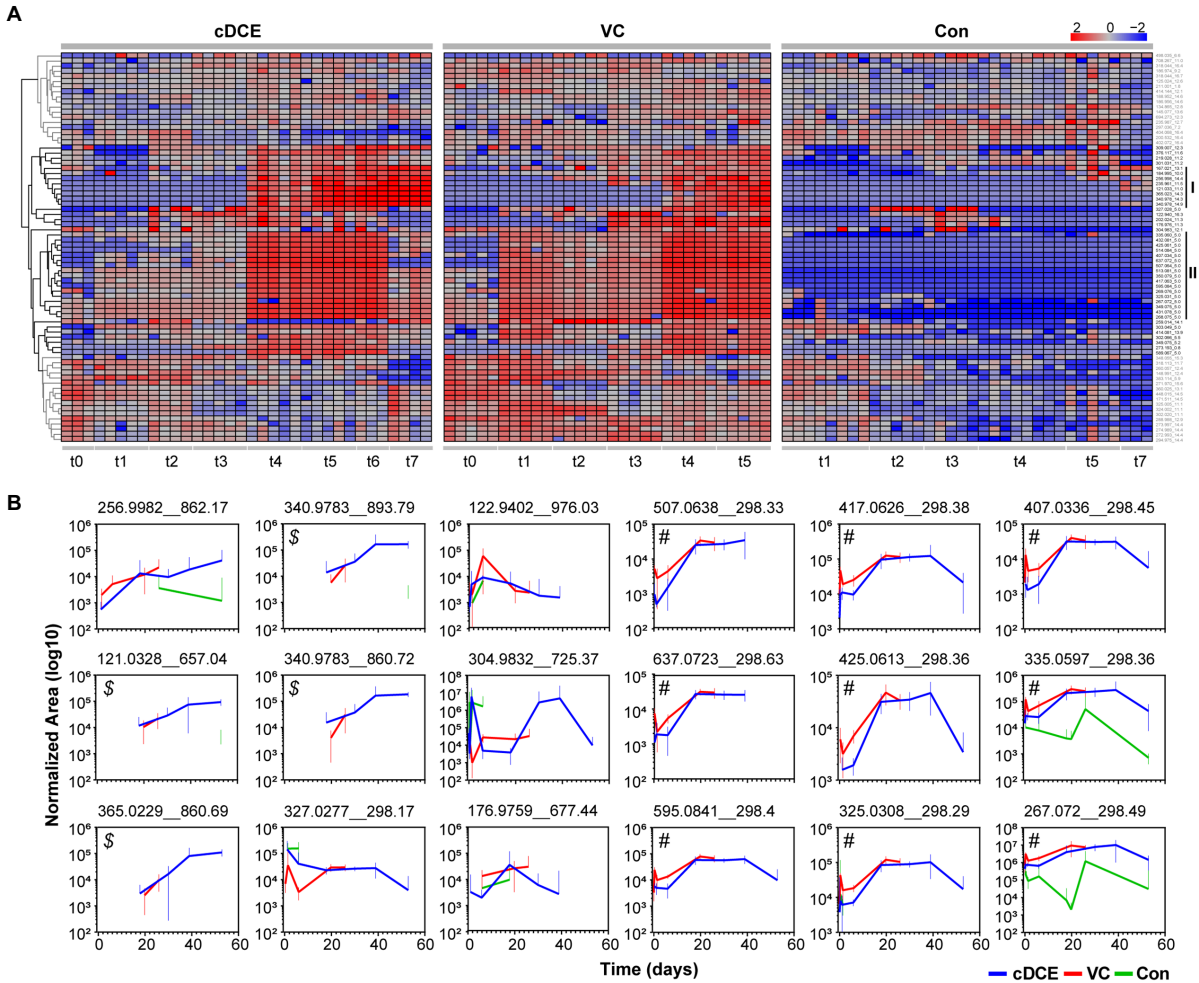
**(A)** Heatmap of 76 significant features determined to be significant by ANOVA analyses. **(B)** Temporal abundances of potential biomarkers identified from clustering analyses for cDCE, VC, and no amendment. Error bars represent 95% confidence intervals of replicate data points. Callouts (*$* and *#*) are used for reference in the results and discussion sections.

**Figure 6:**
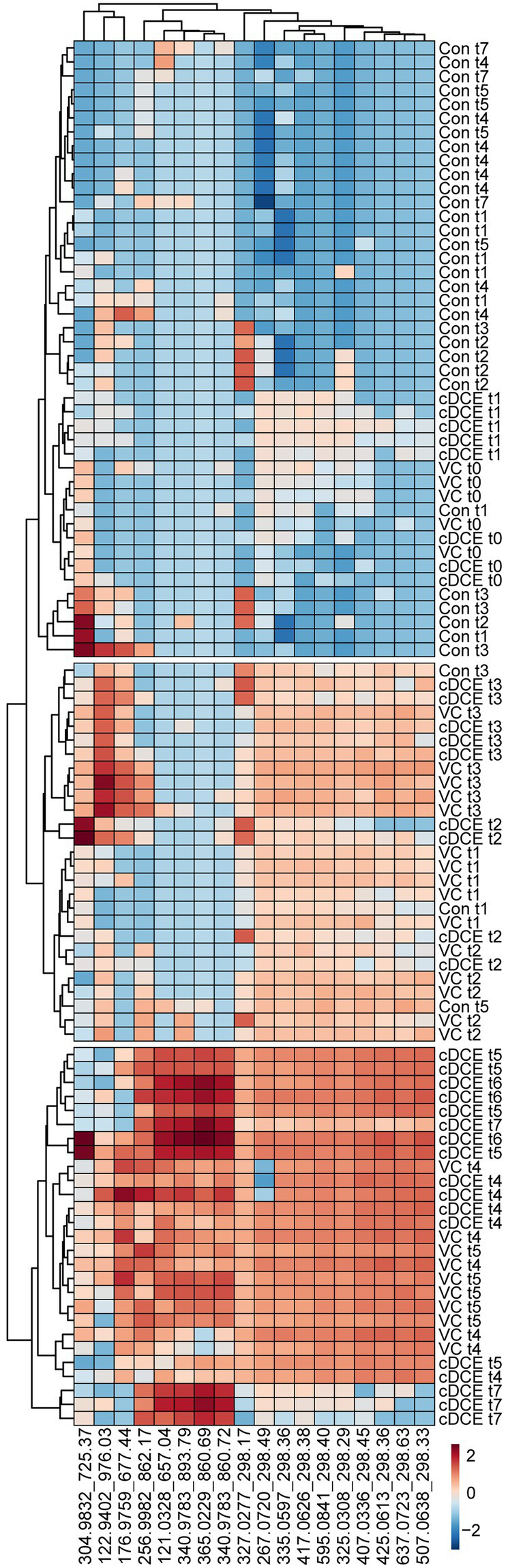
Correlation heatmap of 18 identified biomarkers. Log_10_ transformed metabolomics data are able to differentiate among dechlorination status and time points.

Raw spectra files were converted to a universal format, mzML, using msconvert (Chambers et al., 2012). Feature detection was performed using the online MetaboAnalyst 5.0 platform (Pang et al., 2021). Settings were optimized using a pooled sample for quality control (QC) on the UHPLC-Orbitrap platform. Feature detection and removal from instrument blank was also utilized for optimal feature detection. Detected features were then trimmed using a % coefficient of variation (%CV) of less than 40% for pooled samples. The remaining features were normalized using total bacteria 16S qPCR cell count data and the cell normalized ion intensities were used in all following analyses. *PLSDA Figures.* Normalized features uploaded into the statistical analysis [one-factor] package in the online MetaboAnalyst5.0 platform. Features (40%) were filtered based on interquartile range (IQR) and log transformed prior to PLSDA analysis. Samples were either grouped based on dechlorination activity (active or inactive) or by biological replicates (Figure 2). All samples were included in PLSDA analysis of dechlorination activity (Figure 2A), while only samples within each addition (cDCE or VC) were included in PLSDA analysis of time course (Figure 2B). *ANOVA figures*. Normalized features uploaded into the statistical analysis [metadata table] package in the online MetaboAnalyst5.0 platform. Features (40%) were filtered based on IQR and log transformed prior to two-way ANOVA analysis. Addition of chlorinated electron acceptor (cDCE, VC, or none) and time point (t_0_ – t_7_) were used for ANOVA with interactions. All features that were only significant in time point were removed from *post hoc* analysis. The remaining features were uploaded into the statistical analysis [one-factor] package in the online MetaboAnalyst5.0 platform, grouped by addition, and one-way ANOVA was performed with Fisher LSD *post hoc*. This analysis was repeated for the features removed *via* the IQR data filtration and all significant features from *post hoc* results (Figure 3) were used for the following analyses. *Clustering/heatmap visualization*. Clustering was performed using log 10 and mean-centered ion intensities were calculated using Euclidean distances with complete clustering of rows (features) only. Clustering and heatmap visualization of metadata, which are all measurements other than metabolomics for purposes of this manuscript (Figure 4) was performed using the statistical analysis modules of MetaboAnalyst5.0, no data filtering, and log transformed. Clustering and heatmap visualization for significant features (Figure 5A) was performed using the heatmap package available through the R statistical analysis software. Lastly, metabolite biomarkers (Figure 6) were clustered using the statistical analysis modules of MetaboAnalyst5.0 again with no data filtering, and log transformed.

## Results and discussions

### Metadata-guided sampling intervals

In order to gain a picture of the metabolic changes that differentiate culture showing active versus inactive dechlorination, the metabolome of the commercial bioaugmentation consortium SDC-9™ was captured at representative points in the dechlorination process. It was determined that the dechlorination process was completed after~20 days for VC amended cultures and after~40 days for cDCE amended cultures (Figures 5A,B). Two of five replicates that received VC as electron acceptor did not complete the dechlorination process and were removed from further analysis. The maximum VC concentration for cDCE amended cultures was reached after~4 days (Figure 5A).

Sampling was performed in intervals with the goal to capture unique stages of the SDC-9™ consortium metabolome as the chlorinated electron acceptor(s) were consumed. The sampling time points were selected to capture changes in metabolism, e.g., when either the ratio of chlorinated ethene species had significantly changed or when either cDCE or VC had been completely dechlorinated as judged by disappearance of the molecules from the headspace (Table 1). The qPCR results (Figures 5C,D) revealed that the total bacterial 16S rRNA gene copies reached 5.4 × 10^8^ ± 9.0 × 10^7^ copies/ml and 7.9 × 10^8^ ± 7.3 × 10^7^ copies/ml in cultures amended with VC and cDCE, respectively. In all cultures, lactate concentrations decreased over the first several days, while the total bacteria 16S rRNA gene copies/ml increased until lactate was completely consumed for all cultures after ~6 days. This corresponded to an increase in acetate concentrations to ~7 mM, which remained constant for the remainder of the experiment in all cultures. When lactate was no longer detected, the total bacterial 16S rRNA gene count decreased for both VC (5.4 × 10^8^ ± 9.0 × 10^7^ to 3.0 × 10^8^ ± 6.4 × 10^7^ copies/ml) and cDCE (7.9 × 10^8^ ± 7.3 × 10^7^ to 4.0 × 10^8^ ± 9.7 × 10^7^ copies/ml) treated cultures. This decrease in the number of total bacteria coincided with an increase in *Dhc* 16S rRNA genes from 1.6 × 10^8^ ± 2.7 × 10^7^ to 2.6 × 10^8^ ± 2.6 × 10^7^ copies/ml in cultures with VC and 1.6 × 10^8^ ± 1.3 × 10^7^ to 2.1 × 10^8^ ± 3.3 × 10^7^ copies/ml in cultures with cDCE. In control cultures without chlorinated electron acceptor additions, the *Dhc* 16S rRNA gene copies/ml remained relatively constant over the course of the incubation period (Figure 5E). Due to inconsistent total bacteria numbers from the qPCR results in replicates sampled from VC amended and VC control cultures at t7 (see Table 1), all data from this time point for the VC experiment were removed from further analysis. This variability is likely due to the age of the culture that had been without required substrates for extended periods.

### Measuring the dechlorination metabolome

The untargeted UHPLC-HRMS metabolomics approach measured 7,782–11,104 spectral features per sample after pre-processing using the MetaboAnalyst5.0 (Pang et al., 2021) LC–MS spectra processing module (Table 2). Of these features, only ~2.5% could be identified by matching the mass to charge-retention time (*m/z*-r.t.) pair for the compounds to a library constructed from injected standards. The percent coefficient of variation (%CV) was then used to further trim the number of features for analysis, and only features with less than a 40% CV, as calculated from pooled QC samples were used in further analyses. This left a curated set containing 5,359–6,368 total features (Table 2). As is common in these analyses, the majority of features detected were not accounted for in available metabolite libraries and had unknown structures, and these unknowns are annotated with an *m/z*-r.t. identifier. Although the molecular structures for these compounds are currently elusive, these compounds can still be useful as biomarkers for the dechlorination process due to the reproducibility of their detection. Further, emerging tools such as those available through the GNPS: Global Natural Products Social Molecular Networking (Wang et al., 2016) platform are accelerating the ability to identify novel metabolites, and work is underway to understand further the unique metabolic processes that lead to dechlorination in this system and to determine whether potential biomarkers for this activity can be measured in field samples.

**Table 2:** Average number of detected features from each sample and time point before and after %CV data processing.

Time Point	cDCE		VC		None	
	Total	Trimmed	Total	Trimmed	Total	Trimmed
t0	9,458 ± 502	6,018 ± 139	9,557 ± 468	6,038 ± 97	n/a	n/a
t1	9,725 ± 266	6,116 ± 62	10,222 ± 283	6,222 ± 57	9,687 ± 374	6.089 ± 79
t2	10,925 ± 68	6,335 ± 35	10,986 ± 157	6,322 ± 21	10,883 ± 163	6.312 ± 28
t3	10,766 ± 207	6,338 ± 26	10,905 ± 193	6,337 ± 22	10,568 ± 298	6.287 ± 48
t4	9,515 ± 371	6,027 ± 82	9,939 ± 542	6,169 ± 96	9,444 ± 419	6.030 ± 93
t5	9,178 ± 538	5,895 ± 140	9,202 ± 743	5,862 ± 258	9,036 ± 440	5.899 ± 89
t6	9,373 ± 545	5,934 ± 140	n/a	n/a	n/a	n/a
t7	8,254 ± 431	5,554 ± 140	n/a	n/a	8,435 ± 426	5.646 ± 132

### Multivariate analyses differentiate metabolite profiles in dechlorinating versus non-dechlorinating SDC-9™ cultures

The first statistical tool used to determine whether metabolic differences could be observed during the dechlorination process was PLSDA, which allowed testing the hypothesis that samples that were classified by their dechlorination status as judged by metadata also displayed different metabolic profiles. Cultures that did not receive cDCE or VC, that had not started, or that had completed the reductive dechlorination process were grouped as inactive; and all others were grouped as active. Using the multivariate PLSDA approach, minimal separation was seen between these groups when using only two components; however, when the data were plotted using the top three components, good separation between the active and inactive dechlorination metabolomes emerged (Figure 6A).

Next, samples from only the VC- or cDCE amended cultures were analyzed to determine whether differences in the metabolic profiles could be used to determine the extent of dechlorination within these sample sets. The time points of collection were used to classify the samples for these analyses. It was hypothesized that samples from each time point would group together, and that samples from different time points would show divergence based on the extent of dechlorination. Further, components that showed separation among the time points are due to the variability in the metabolite profiles; and the top two components explained ~57% of the variation for cultures that were grown with cDCE and ~ 60% of the variation for cultures that were grown with VC (Figure 6B). Interestingly, the samples seemed to organize based on the extent of dechlorination, which correlated with time. Component 1 for both VC and cDCE amended samples shows separation into three time periods with t2 being the divergence point for VC and t3 for cDCE. These specific samples were collected 1.5–2 days after the start of VC dechlorination indicating there may be metabolites specifically associated with the onset of VC dechlorination. The metabolomes from the beginning stages of dechlorination, t0 and t1, cluster closely, but remain mostly separated, for both VC and cDCE cultures. Next, it was observed that sampling time point t2 cDCE formed a tight cluster distinct from t0 and t1 along the component 2 axis, but not the component 1 axis. This observation indicated that samples collected during the early stages of cDCE dechlorination have a unique metabolic profile. As dechlorination reached completion in the VC amended samples, t3 separated from t2 on the component 1 axis, while t4 and t5 formed a larger cluster that was separated from t3 on the component 2 axis. These data indicated that following consumption of most chlorinated electron acceptor, the metabolic profiles for near complete dechlorination could still be distinguished from those associated with complete dechlorination. As dechlorination progressed in cDCE-amended cultures, the remaining four time points, t4-t7, formed a larger cluster that was distinct from t0-t3 measurements along the component 1 axis indicating a third time period or phase during the dechlorination process, in which the consumption of cDCE was complete but VC dechlorination was still ongoing. Dechlorination was complete at t7 for cDCE and t5 for VC, and the clusters for these samples started to diverge along both component axes indicating that the metabolic profiles are no longer consistent with those observed during active dechlorination.

### Assessing the utility of volatile organic compound concentrations and bacterial 16S rRNA gene enumeration metadata for determining dechlorination status

In order to determine whether metabolomics can predict dechlorination status, the metabolome was compared to a set of MBTs commonly used in the field. A correlation heatmap of the metadata, which used concentrations for all measured VOCs and 16S rRNA gene qPCR results, was created and the first five nodes are labeled 1 to 3 (Figure 3). Early stage dechlorination samples clustered separately from all other samples (clusters 1 and 3a), however, cluster 1 was noted to be most divergent from all other samples taken, likely because they were the only ones with high concentration of cDCE (samples cDCE t0-t2). Similarly, time points with high concentrations of only VC (samples VC t0-t1) grouped together in cluster 3a. Samples that were undergoing active dechlorination (VC t2-t3 and cDCE t3 samples plus one cDCE t4 sample) could not be differentiated the from controls taken at similar time points (Con t2-t3) using the metadata collected for this analysis as seen in cluster 2b. This is likely due to the fact that the clustering for these samples is strongly influenced by total bacterial numbers as most other measurements were not sufficiently different to differentiate among sample types. Samples at or near the end of the dechlorination process (VC t4-t5 and cDCE t4-t5) could be differentiated from controls using the metadata, and they grouped in cluster 2a. Finally, it was noted that all of the later stage control samples clustered together and separately from all amended samples except for those from the final time point for the cDCE cultures (cDCE t7), which were taken 14 days after all chlorinated electron acceptors had been consumed. Although several subclusters were observed, e.g., all VC t2 samples grouped separately after two more nodes within cluster 2b, the degree of differentiation is not as large as for the larger clusters, and it is unlikely that the nuances leading to these subclusters would be observable outside of controlled laboratory conditions due to the lack of controls and the expected higher variability of field data. It should be noted that the initial *Dhc* titer used in this study was high, which could explain the lack of correlation between dechlorination activity and total bacterial 16 S rRNA gene copy numbers. This observation highlights the benefit of having complementary validated techniques to monitor a process as multiple lines of evidence are often needed.

### Probing the metabolome for small molecule biomarkers for the reductive dechlorination process

After determining that the metabolome could be used to monitor the dechlorination status of the SDC-9™ cultures, analyses were conducted to explore whether a discrete set of small molecule biomarkers that were indicative of organohalide respiration with cDCE and/or VC as electron acceptor(s) could be identified. To differentiate between features that might be associated with the dechlorination process from those only correlated to bacterial growth not directly linked to organohalide respiration, the metabolome features were subjected to a two-way ANOVA, in which samples from the cultures were grouped by type of addition (cDCE, VC, or none) and the addition by time interactions of the metabolome with time points t0-t7 were assessed (Figure 1). First, the set of 14,130 metabolite features was reduced to 6,574 features by selecting only those that had a CV <40%. Due to limitations within MetaboAnalyst online that limit the total number of features to fewer than the 6,547 that remained in this dataset, interquartile range (IQR) data filtering was used to segregate 40% of the total metabolite features into a set of 2,630 features. Both the IQR (2,630 metabolites) and outer quartile (3,944 metabolites) data sets were analyzed separately using two-way ANOVA to further reduce the number of features before final analyses with one-way ANOVA between subjects to finalize a list of candidate biomarkers. Combined, the two-way ANOVA analyses removed a total of 2,528 features that were either considered non-significant or significant by only time and not type of electron acceptor added. The remaining features (1,599 from the IQR and 3,944 from the outer quartile set) were grouped only by addition (cDCE, VC, or none), and each set was analyzed separately *via* a one-way ANOVA with Fishers LSD *post hoc* analysis (Figure 1). *Post hoc* analyses that placed cultures with no added chlorinated ethenes (VC-none and cDCE-none) in the same direction yielded 76 features in total. This set of features contains the most likely candidates for biomarker potential and was further evaluated. Of the 14,130 features initially detected, only 15 were unique across the time course for VC and cDCE amended cultures and absent in the controls, and 12 (out of 15) of these features were found to be statistically significant among treatments and time points. Further, there were no unique features detected in the control cultures indicating that the metabolism used for the dechlorination process does not downregulate other metabolic functions within the consortium.

This set of 76 features that were statistically correlated to active dechlorination was further analyzed to determine how the concentrations of these molecules related to the phases of the reductive dechlorination process. First, the median area counts for each metabolite feature were subtracted from the absolute area counts of that metabolite for each sample, and the resulting value was log10 normalized before being visualized in a heatmap (Figure 2A). Two distinct clusters of features that are indicative of dechlorination activity were observed (highlighted with black as clusters I and II in Figure 2A), and both have several features not reproducibly detected in the control cultures. Metabolites in cluster I generally increased during the later time points, while the features in cluster II increased over the early time points before decreasing near the end of the experiment for cDCE amended cultures (see cDCE t7). This decrease was not observed in VC amended cultures; however, this could be because the last time point was taken ~20 days earlier at the completion of VC dechlorination, which is much later than the final time point for the cDCE amended cultures. Of the initial 76 metabolite features included in the heatmap, only 41 were found in clusters I and II and utilized for further evaluation. After manual inspection of the peaks for the 41 remaining compounds, it was noted that several of the features arose from isotopes and adducts, which left a total of 18 potential biomarkers for reductive dechlorination once the duplicate measurements for replicate metabolite were removed.

MetaboAnalyst5.0 was only able to annotate two of the 18 features based on the exact mass of these molecules: *m/z* 121.0328 was annotated as thiodiglycol, and *m/z* 122.9402 as 1,2,4-trithiolane. Even though only two of the metabolite features have annotated structures, this set of 18 metabolites is still useful as biomarkers for the dechlorination process, and further experiments are underway to determine the structures for these compounds. These metabolites were plotted over time and per condition (Figure 2B), and a set of four features (*$* in Figure 2B) began to appear as the consumption of the initial chlorinated ethene was nearly complete for both VC and cDCE amended cultures, and these features increased in abundance until the end of the incubation periods. Another set of nine features (*#* in Figure 2B) had concentrations that followed the *Dhc* 16S rRNA gene abundances (monitored by qPCR), and seven of these molecules were only detected in cultures treated with VC or cDCE. Together, these results indicate that there are unique metabolites that are directly associated with both dechlorination activity and the *Dhc* abundance within the SDC-9™ consortium. Further investigations are warranted to determine whether these compounds can be reproducibly linked with SDC-9™ reductive dechlorination activity in laboratory cultures and at field sites where bioaugmentation with consortium SDC-9™ supports remediation efforts.

Untargeted metabolome analysis of SDC-9™ cultures amended with cDCE or VC as electron acceptors revealed metabolite patterns that distinguish actively dechlorinating cultures from non-dechlorinating cultures (i.e., cultures that had consumed all chlorinated electron acceptors or had not initiated reductive dechlorination activity). A set of 18 small molecule biomarkers that correlated with dechlorination status and/or *Dhc* abundance were identified, and these molecules revealed a strong correlation with dechlorination status in laboratory SDC-9™ cultures indicating their potential value as process-specific biomarkers (Figure 4). Cluster analyses organized the metabolome into three groups according to the extent of dechlorination, and four metabolite features were detected in increasing temporal abundance in all samples collected during time points t4-t7 from both VC and cDCE amended cultures (*$* in Figure 2B). Taken together, these observations suggest that potential metabolome biomarkers for measuring dechlorination activity exist. This study illustrates the potential utility of metabolomics as either a complementary or stand-alone MBT for monitoring the progress of bioremediation at sites impacted by chlorinated ethenes. While demonstration and validation work is needed to ground truth the utility of this technique for field samples, the data presented herein are a crucial first step in the MBT development process and may also lead to a more complete understanding of the metabolic processes that govern reductive dechlorination.

## Data availability statement

The datasets generated for this study can be found in the MetaboLights (Haug et al., 2019) database with the identifier MTBLS5219 (www.ebi.ac.uk/metabolights/MTBLS5219).

## Author contributions

AM, SC, and FL conceptualized the study. AM, YX, SC, and FL designed the experiments. AM performed the laboratory experiments with assistance from YX and FM. AM performed the analyses. AM, SC, and FL discussed data and interpretation. AM visualized the results. AM, SC, and FL wrote the manuscript. MM contributed to manuscript improvements. All authors contributed to the article and approved the submitted version.

## Funding

This work was funded by the US Army Corps of Engineers (W912DW-17-P00) and NIH SBIR R43 (ES030669-01).

## Conflict of interest

The authors declare that the research was conducted in the absence of any commercial or financial relationships that could be construed as a potential conflict of interest.

## Publisher’s note

All claims expressed in this article are solely those of the authors and do not necessarily represent those of their affiliated organizations, or those of the publisher, the editors and the reviewers. Any product that may be evaluated in this article, or claim that may be made by its manufacturer, is not guaranteed or endorsed by the publisher.
